# Predicting the risk of depression in older adults with disability using machine learning: an analysis based on CHARLS data

**DOI:** 10.3389/frai.2025.1624171

**Published:** 2025-07-02

**Authors:** Tongtong Jin, Ayitijiang· Halili

**Affiliations:** ^1^School of Law, Shanxi University of Finance and Economics, Taiyuan, China; ^2^College of Public Management (Law), Xinjiang Agricultural University, Urumqi, China

**Keywords:** disabled older adults, depression, risk prediction, machine learning, CHARLS, mental health LR, HistGBM, MLP

## Abstract

**Background:**

The advancement of artificial intelligence technologies has opened new avenues for depression prevention and management in older adults with disability (defined by basic or instrumental activities of daily living, BADL/IADL). This study systematically developed machine learning (ML) models to predict depression risk in disabled elderly individuals using longitudinal data from the China Health and Retirement Longitudinal Study (CHARLS), providing a potentially generalizable tool for early screening.

**Methods:**

This study utilized longitudinal data from the CHARLS 2011–2015 cohort. A three-stage serial consensus approach feature selection framework (LASSO, Elastic Net, and Boruta) was employed to identify 21 robust predictors from 74 candidate variables. Ten ML algorithms were evaluated: LR, HistGBM, MLP, XGBoost, bagging, DT, LightGBM, RF, SVM, and CatBoost. Temporal external validation was performed using an independent 2018–2020 cohort to assess model generalizability. Performance was comprehensively evaluated using accuracy, AUC, F1-score, precision, and recall metrics. The SHAP framework was employed to interpret feature contribution mechanisms.

**Results:**

Results demonstrated that the HistGBM model achieved optimal overall performance on the testing sets (AUC = 0.779, F1-score = 0.735, accuracy = 0.713), with only an 8.5% AUC difference between training and testing sets and a 10% difference between external validation and testing sets, indicating temporal stability. SHAP interpretability analysis revealed that sleep time (mean SHAP value = 0.344) in the health behavior domain and life satisfaction (0.339) and episodic memory (0.220) in the subjective perception domain contributed more significantly to prediction than traditional biomedical indicators.

**Conclusion:**

This study developed an AI-based tool for depression risk assessment in older adults with disability through a multi-stage feature selection process and a temporal external validation framework. These findings provide a practical screening instrument and a methodological reference for implementing AI technologies in geriatric mental health applications, thereby facilitating clinical translation of predictive analytics in this field.

## 1 Introduction

The comorbidity of disability and depression among older adults is a growing concern. Disability refers to a state of limited activities of daily living due to physical or mental impairments. At the same time, depression is a neuropsychiatric condition principally manifested through sustained affective dysregulation, significantly compromising physiological functioning and social adaptability (Kupferberg et al., 2016). A well-established bidirectional association exists between disability and depression (Zhou et al., 2024; Zhu et al., 2024). Disability contributes to depression through loss of social roles and restricted mobility, whereas depression exacerbates functional decline by reducing rehabilitation adherence and impairing immune function. Epidemiological studies indicate that the global prevalence of major depressive disorder in older adults is approximately 13.3% (Abdoli et al., 2022), with disabled elders exhibiting significantly higher risks than their unimpaired counterparts (Asdaq SMB et al., 2024). In China, the prevalence of geriatric depression reaches 34.1% (Feng et al., 2021), with rural areas demonstrating elevated vulnerability due to limited healthcare access and weaker familial support systems. This vicious cycle between disability and depression not only accelerates individual functional deterioration but also imposes substantial healthcare burdens and societal costs (Wang et al., 2023; Ni et al., 2024).

Previous studies have predominantly employed cross-sectional designs and conventional statistical approaches (e.g., logistic regression, fixed-effects models) to identify risk factors. Regarding risk association validation, Mu et al. (2022) demonstrated through binary logistic regression that individuals with disability exhibit significantly elevated risks of depressive symptoms. Using multivariate logistic regression, Yan et al. (2023) further revealed urban–rural differential effects in the disability-depression association. In terms of disease trajectory research, Tian et al. (2022) found that individuals with disability were more likely to follow trajectories of worsening depressive symptoms. Musliner et al. (2016) associated prevalence rates, Çağan and Ünsal (2014) reported a 57.8% depression rate among disabled individuals, while McGillivray and McCabe (2007) documented a 39.1% depression prevalence among those with mild-to-moderate intellectual disabilities. Individual emotional states, life satisfaction, self-rated health, and social support systems have been systematically validated as critical predictors (Turner and Noh, 1988; Song et al., 2023). However, traditional linear models demonstrate limited capacity in analyzing high-dimensional nonlinear relationships, and their static data frameworks fail to capture the temporal cumulative effects of risk factors.

In summary, as shown in Table 1, existing studies exhibit three major limitations: (1) Design dimension: Prior studies predominantly rely on cross-sectional data, failing to capture the temporal cumulative effects of risk factors (e.g., the progressive impact of disability deterioration on depression). (2) Methodological dimension: Although conventional linear models (e.g., logistic regression) can validate risk associations, they struggle to handle high-dimensional nonlinear relationships. In contrast, ML algorithms significantly enhance predictive performance by extracting feature interactions and identifying temporal patterns. (3) Feature dimension: Existing research excessively focuses on physiological indicators (e.g., disease burden, functional impairment) while neglecting the contributions of subjective cognition (e.g., life satisfaction) and health behaviors (e.g., sleep). This study advances beyond conventional paradigms by integrating a longitudinal design, ML approaches, and a multidimensional feature structure to address these limitations. Specifically, we utilize multi-wave longitudinal data from CHARLS (2011–2020) and incorporate a temporal external validation framework (using an independent 2018–2020 cohort) to track the evolving trajectories of disability and depression dynamically. We systematically compare 10 ML algorithms and introduce the SHapley Additive exPlanations (SHAP) interpretability framework to balance predictive accuracy with mechanistic insights. Furthermore, we construct a multidimensional feature matrix and employ a three-stage serial consensus feature selection (LASSO, Elastic Net, and Boruta), demonstrating that subjective perceptions (SHAP value: life satisfaction = 0.339) and health behaviors (sleep time = 0.344) exhibit stronger predictive power than conventional biomedical indicators. This integrative approach not only overcomes prior methodological constraints but also provides a robust, interpretable, and clinically actionable framework for depression risk stratification in older adults with disabilities.

**Table 1:** Paradigm comparison between this study and previous depression prediction studies.

Dimension	Previous mainstream research	The innovation point of this study
Design	Cross-sectional data	Multi-wave longitudinal data + temporal external verification
Method	Traditional statistical models	Comparison of 10 ML Algorithms + SHAP interpretation
Feature	Physiological indicators	Integration of subjective perception/health behavior/physiological multidimensional characteristics

Although machine learning (ML) offers innovative solutions to address these limitations (Zhao et al., 2025; Fan et al., 2025), recent studies based on CHARLS data still exhibit notable shortcomings: (1) Overreliance on a single algorithm for feature selection. For instance, Huang et al. (2025) employed LASSO exclusively for feature selection in cardiovascular disease risk prediction among middle-aged and older adults (Huang et al., 2025), which may inadequately address the challenges of high-dimensional feature collinearity and stability; (2) Insufficient temporal external validation in the evaluation framework. As demonstrated by Chu et al. (2025), the disability prediction model for older adults lacked external validation (Chu et al., 2025), potentially compromising the generalizability of the findings.

ML offers innovative solutions to overcome these methodological limitations (Vu et al., 2025; Ai et al., 2024). Compared to conventional approaches, ML demonstrates superior predictive performance through its capacity for feature interaction mining and temporal pattern recognition (Nickson et al., 2023). Xin and Ren (2022) developed random forest models to predict disability risk in urban and rural populations, achieving AUC values of 0.71 and 0.78, respectively. In a systematic comparison, Hong et al. (2025) demonstrated that XGBoost models exhibited excellent performance in training sets (AUC = 0.76), while logistic regression models performed well in validation sets (AUC = 0.73). Handing et al. (2022) employed random forest analysis and identified social isolation and self-rated health as significant determinants of depression. Despite these advancements, few studies in China have utilized longitudinal data and multiple ML algorithms to construct risk prediction models specifically for depressive disorders within geriatric populations with functional limitations (Hong et al., 2025). Furthermore, methodological weaknesses in validation frameworks among existing studies may compromise the reliability of findings (Huang et al., 2025; Han and Wang, 2023).

This study utilized multi-wave data (2011–2020) from the China Health and Retirement Longitudinal Study (CHARLS) to construct a predictive computational framework for geriatric populations with functional limitations. We integrated three waves of panel data (2011–2015) to construct a comprehensive feature matrix encompassing baseline characteristics, disease profiles, and disability progression patterns. A three-stage serial consensus approach was utilized to identify robust predictors combining elastic net regularization, least absolute shrinkage and selection operator (LASSO), and Boruta algorithms. We identified 21 robust predictors from 74 candidate variables. A temporal external validation strategy was implemented using an independent 2018–2020 cohort to systematically evaluate the cross-temporal stability of 10 ML models, including HistGBM. The study aims to provide a high-accuracy tool for early identification of depression risk in disabled older populations and establish evidence-based priorities for psychosocial interventions.

## 2 Methods

### 2.1 Data sources and research design

**Figure 1:**
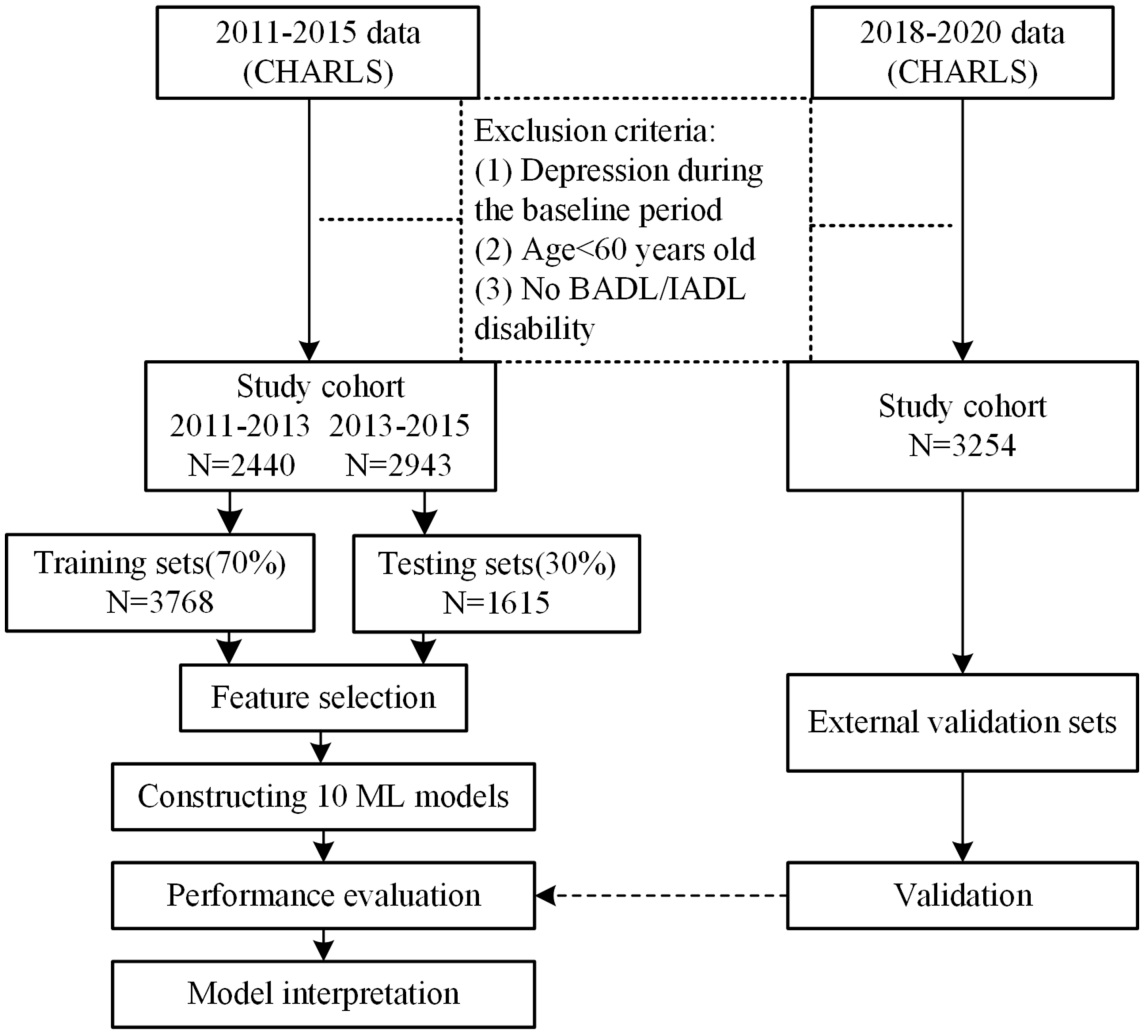
Research flowchart.

This study utilized data from the CHARLS, which implements multistage stratified sampling with probability-proportional-to-size weighting based on demographic stratification. The survey encompasses 150 county-level units across 28 provincial administrative regions in China. The baseline survey was conducted in 2011, with follow-up waves completed in 2013, 2015, 2018, and 2020, collecting comprehensive data on demographic characteristics, socioeconomic status, health behaviors, and medical history. The study protocol obtained ethical certification from Peking University’s Biomedical Ethics Committee (Approval ID: IRB00001052-11015). Sample selection followed three inclusion criteria: (1) age ≥60 years at baseline; (2) exclusion of individuals with pre-existing depression diagnosis or without basic or instrumental activities of daily living (BADL/IADL) disability at baseline; (3) completion of at least two consecutive follow-up assessments. Through integration of baseline (2011–2013, *N* = 2,440) and follow-up (2013–2015, *N* = 2,943) data, we constructed a longitudinal panel dataset containing 5,383 observations (2011–2015). The dataset was partitioned using stratified random sampling, allocating samples in a 7:3 ratio to training (*N* = 3,768) and testing (*N* = 1,615) sets. An independent 2018–2020 follow-up cohort (*N* = 3,254) served as the external validation set. The study flowchart is presented in Figure 1.

### 2.2 Variable definitions and measurements

Depression was assessed using the 10-item Center for Epidemiological Studies Depression Scale (CESD-10), a widely validated screening tool for depressive symptoms in older adults (Zhang et al., 2024; Wang et al., 2023). The scale demonstrates good reliability and validity (Chen and Mui, 2014; Cheng and Chan, 2005). In CHARLS, the CESD-10 evaluates the frequency of 10 symptoms experienced in the past week: feeling bothered, trouble concentrating, feeling depressed, difficulty in doing things, feeling hopeful about the future (reverse-coded), feeling fearful, restless sleep, feeling happy (reverse-coded), feeling lonely, feeling unable to carry on. Each item is scored from 0 to 3, yielding a total score ranging from 0 to 30. Following established international criteria, a score ≥10 was used to define clinically significant depressive symptoms.

Disability in this study specifically refers to functional limitations in BADL or IADL, consistent with geriatric assessment standards (Katz et al., 1963; Lawton and Brody, 1969). BADL evaluates six fundamental self-care functions (dressing, bathing, eating, bed transferring, toileting, and continence control). IADL assesses five complex daily living skills (housekeeping, cooking, shopping, medication management, and financial management), where each item was scored 1 for inability to perform independently or 0 otherwise, resulting in total score ranges of 0–6 for BADL and 0–5 for IADL. The BADL/IADL-based criteria were applied during data screening to select eligible participants with BADL≥2 or IADL≥2 scores. This operational definition excludes sensory or cognitive disabilities alone, ensuring a homogeneous cohort with physical functional impairments.

The definitions and measurement approaches of covariates encompassed four domains: (1) demographic characteristics (gender, age, registered residence, educational level, marital status, number of children, and region); (2) health behaviors, including chronic disease history (14 conditions such as hypertension and diabetes), sensory functions (visual, auditory, and oral health assessed through assistive device use, functional scores, and tooth loss status), bodily pain, sleep time, physical activity intensity, social engagement, and lifestyle factors; (3) subjective perceptions comprising episodic memory, cognitive ability, life satisfaction, and self-rated health; (4) health care and insurance, incorporating health insurance type, healthcare utilization (frequency, duration, and costs of inpatient and outpatient services), as well as pension status, with detailed variable specifications and coding schemes provided in Supplementary Table 1.

### 2.3 Data preprocessing and feature selection

Data preprocessing included four key steps: (1) Outlier handling: We applied the interquartile range (IQR) method to detect and truncate outliers for all continuous variables. Values beyond ±1.5IQR of the 25th-75th percentile range were clipped to the lower/upper bounds. This mitigated the impact of extreme values on tree-based models while preserving data distribution integrity. (2) One-hot encoding: Categorical variables (e.g., gender, region) were converted into binary dummy variables to avoid misinterpreting ordinal relationships. (3) Normalization: Continuous features were standardized using z-score normalization (mean = 0, variance = 1) to enhance convergence speed for linear models. (4) Missing value imputation: Among 78 candidate variables, 4 variables (5.13%) with missing rates >30% were excluded; (5) Missing value imputation: The remaining 74 variables had an average missing rate of 8.27% (range: 0.09–25.18%). A total of 15,918 missing records (8.82% of total training observations) were iteratively imputed using the MissForest algorithm. (6) Class imbalance adjustment: We implemented the SMOTE-Tomek hybrid sampling technique, combining synthetic minority oversampling (SMOTE) with Tomek links under sampling. This approach effectively enhanced the model’s sensitivity in detecting depression risk and improved clinical utility by generating synthetic samples.

We refer to existing studies for feature selection (Huang et al., 2025; Zheng et al., 2024; Zhu et al., 2025). A three-stage serial consensus approach was utilized to identify robust predictors in this study. This serial consensus approach integrates complementary strengths of distinct selection paradigms. (1) LASSO (L1 regularization): Efficiently screens out zero-importance features (73 variables) by imposing sparsity constraints. Although its linearity assumption may oversimplify relationships, it serves as a high-recall initial filter. (2) Elastic Net (L1 + L2 regularization): Reduces multicollinearity-induced instability by retaining correlated but biologically plausible features. The *α* = 0.5 setting balances sparsity and grouping effects, mitigating LASSO’s limitation in correlated feature selection, retaining 42 stable features. (3) The Elastic Net output variables were fed into the Boruta algorithm, which identified 28 significant predictors by comparing random forest importance scores with shadow variables (*p* < 0.01). To ensure reproducibility, a random seed (random_state = 42) was set for both the MissForest imputation and Boruta’s shadow variable generation.

**Figure 2:**
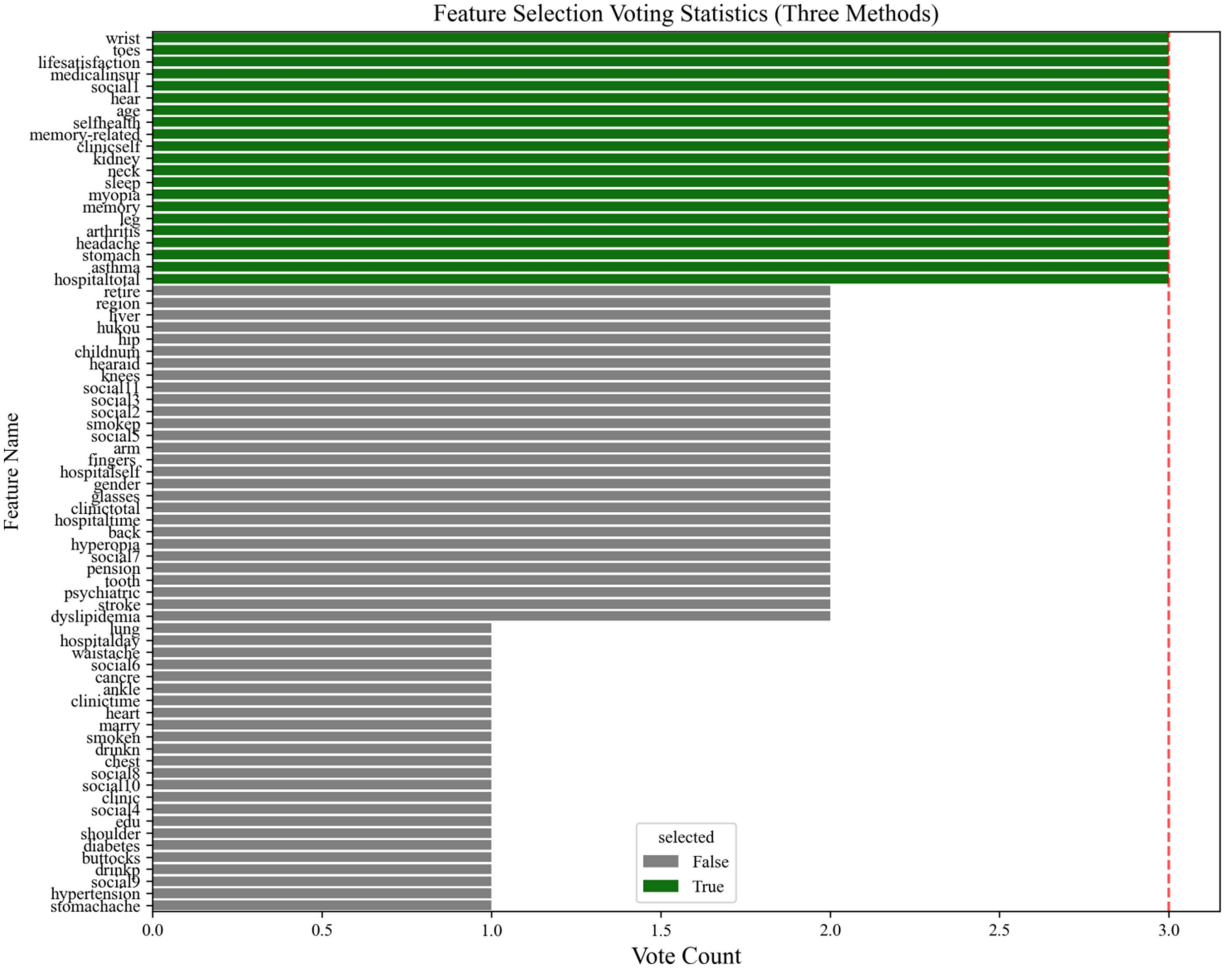
Feature selection results using three methods (LASSO, Elastic Net, Boruta).

Integration of feature selection results via a strict intersection strategy. The three feature selection outcomes were consolidated through a stringent intersection strategy. Specifically, we quantified the selection frequency of each variable across LASSO, elastic net, and Boruta algorithms, retaining only variables unanimously selected by all three methods (i.e., frequency ≥3). This approach yielded 21 high-confidence predictors, including age, self-rated health, arthritis, renal disease, stomach, asthma, memory-related disorders, observe the situation up close, hearing ability, self-reported pain in head, wrist, leg, toes, neck, sleep time, social activities, episodic memory, life satisfaction, medical insurance types, hospitalization expenses (total expenses), outpatient expenses (out of pocket expenses) (as shown in Figure 2). Compared to individual methods, this strategy significantly enhanced feature stability.

### 2.4 Model construction and performance evaluation

Ten ML algorithms were implemented, including logistic regression (LR), support vector machine (SVM), extreme gradient boosting (XGBoost), light gradient boosting machine (LightGBM), categorical boosting (CatBoost), random forest (RF), bootstrap aggregating (Bagging), histogram-based gradient boosting machine (HistGBM), multilayer perceptron (MLP), and decision tree (DT). To optimize model generalizability, hyperparameter tuning was performed using grid search with 3-fold stratified cross-validation (specific hyperparameter configurations are provided in Supplementary Table 2). Model performance was comprehensively evaluated through five metrics: (1) the area under the receiver operating characteristic curve (AUC), measuring the model’s ability to discriminate between positive and negative cases (Formulas 1–3); (2) accuracy, representing the proportion of correctly classified samples (Formula 4); (3) precision, indicating the ratio of true positives among all predicted positives (Formula 5); (4) recall, reflecting the model’s capacity to identify actual positive cases (Formula 6); and (5) the F1-score, the harmonic mean of precision and recall, which provides a balanced assessment of the model’s performance on the positive class (Formula 7). The mathematical formulations were derived from established methodologies (Theerthagiri, 2022; Yang and Ying, 2022).


(1) $$\mathrm{TPR}=\frac{\mathrm{TP}}{\mathrm{TP}+\mathrm{FN}}$$

(2) $$\mathrm{FPR}=\frac{\mathrm{FP}}{\mathrm{FP}+\mathrm{TN}}$$

(3) $$\mathrm{AUC}=\int_{0}^{1}\;\mathrm{TPR}(\mathrm{FPR})d(\mathrm{FPR})$$

(4) $$\text{Accuracy}=\frac{\mathrm{TN}+\mathrm{TP}}{\mathrm{FP}+\mathrm{TN}+\mathrm{TP}+\mathrm{FN}}$$

(5) $$\text{Prescision}=\frac{\mathrm{TP}}{\mathrm{TP}+\mathrm{FP}}$$

(6) $$\text{Recall}=\frac{\mathrm{TP}}{\mathrm{TP}+\mathrm{FN}}$$

(7) $$F1=2\times \frac{\text{precision}\times \text{Recall}}{\text{Precision}+\text{Recall}}$$

In these formulations, TPR denotes the true positive rate, FPR represents the false positive rate, TP indicates true positives, FP signifies false positives, TN refers to true negatives, and FN stands for false negatives.

### 2.5 Statistical analysis

Statistical analyses were performed using Stata 18.0 for data description and Python 3.13 for subsequent modeling. Continuous variables were characterized differentially based on their distribution: normally distributed variables were presented as mean ± standard deviation, while non-normally distributed variables were summarized using median and interquartile range, with normality assessed via the Shapiro–Wilk test. Categorical data were expressed as cardinality measures (absolute frequencies) with proportional composition. The statistical significance threshold was set at *p* < 0.05 for all analyses.

## 3 Results

### 3.1 Baseline characteristic

The analysis included samples from the training sets (*N* = 3,768), testing sets (*N* = 1,615), and external validation sets (*N* = 3,254). Table 2 summarizes the baseline demographic characteristics, disease status, and depression status across the three cohorts. The median ages were 71, 71, and 72 years in the training, testing, and validation sets, respectively, with statistically significant inter-group differences (all *p* < 0.05). In terms of gender, the proportion of females is similar in the training sets (57.94%), testing sets (57.28%), and external validation sets (60.97%). There was no statistically significant difference in gender distribution between groups (all *p* > 0.05), indicating that the gender ratio remained balanced in the data partitioning. In terms of marital status, the married group accounts for 70.25, 70.59, and 69.61% of the three groups, respectively, which is much higher than the unmarried group. In terms of registered residence, the proportion of rural registered residence registration slightly decreased in training sets (79.91%), testing sets (79.57%), and external verification sets (78.83%), but all exceeded 78%. There was a significant difference in the distribution of registered residence among groups (*p* < 0.05). The proportion of “3 or more children” in the three groups was 72.24, 73.32, and 66.04%, respectively, with a significant decrease in the proportion of external validation sets. There were significant differences in distribution between groups (all *p* < 0.05). There was no significant difference in regional distribution between groups (all *p* > 0.05). In terms of education level, the proportion of people who have not received formal education gradually decreased in the training sets (68.21%), testing sets (69.05%), and external validation sets (65.43%), while the proportion of high school and above education increased from 4.03 to 5.53%. There was a significant difference between the groups (all *p* < 0.05). In terms of disease characteristics, there was no significant difference (*p* > 0.05) in the prevalence of memory-related diseases and stroke diseases among the training sets, testing sets, and external validation sets. The incidence of heart disease was significant in the training and testing sets (*p* < 0.05), but not significant in the validation sets (*p* > 0.05). The incidence of arthritis disease remained stable among the three groups (56.22–56.61%), with no significant difference between the groups (*p* > 0.05). The proportion of depression showed a significant increasing trend among the training sets (56.32%), testing sets (56.35%), and validation sets (64.20%), with no significant difference (*p* > 0.05).

**Table 2:** Baseline features of training, testing, and validation sets.

Characteristics	Training sets		Testing sets		External validation sets	
	*N* = 3,768	*p*-value	*N* = 1,615	*p*-value	*N* = 3,254	*p*-value
Demographic characteristics						
Age (years)	71 [65–77]	*p* < 0.05	71 [65–77]	*p* < 0.05	72 [66–79]	*p* < 0.05
Gender, *n* (%)						
Female	2,183 (57.94)	*p* > 0.05	925 (57.28)	*p* > 0.05	1984 (60.97)	*p* > 0.05
Male	1,585 (42.06)		690 (42.72)		1,270 (39.03)	
Marital status, n (%)						
Married	2,647 (70.25)	*p* > 0.05	1,140 (70.59)	*p* < 0.05	2,265 (69.61)	*p* > 0.05
Unmarried	1,121 (29.75)		475 (29.41)		989 (30.39)	
Registered residence, n (%)						
Urban	757 (20.09)	*p* < 0.05	330 (20.43)	*p* < 0.05	689 (21.17)	*p* < 0.05
Rural	3,011 (79.91)		1,285 (79.57)		2,565 (78.83)	
Number of children, *n* (%)						
0 children	111 (2.95)	*p* < 0.05	38 (2.35)	*p* < 0.05	51 (1.57)	*p* < 0.05
1 child	224 (5.94)		99 (6.13)		226 (6.94)	
2 children	711 (18.87)		294 (18.20)		828 (25.45)	
3 children and above	2,722 (72.24)		1,184 (73.32)		2,149 (66.04)	
Region, *n* (%)						
Eastern	1,066 (28.29)	*p* > 0.05	450 (27.86)	*p* > 0.05	1,007 (30.95)	*p* > 0.05
Central	1,326 (35.19)		571 (35.36)		1,131 (34.76)	
Western	1,376 (36.52)		594 (36.78)		1,116 (34.29)	
Educational level, *n* (%)						
Illiteracy	2,570 (68.21)	*p* < 0.05	1,115 (69.05)	*p* < 0.05	2,129 (65.43)	*p* < 0.05
Elementary schools	740 (19.64)		308 (19.07)		603 (18.53)	
Junior high schools	306 (8.12)		127 (7.86)		342 (10.51)	
High school and above	152 (4.03)		65 (4.02)		180 (5.53)	
Disease history						
History of memory-related diseases, *n* (%)						
No	3,175(84.26)	*p* < 0.05	1,377(85.26)	*p* < 0.05	2,760(84.82)	*p* < 0.05
Yes	593(15.74)		238(14.74)		494(15.18)	
History of heart disease, *n* (%)						
No	2,667(70.78)	*p* < 0.05	1,159(71.76)	*p* < 0.05	2,102(64.60)	*p* > 0.05
Yes	1,101(29.22)		456(28.24)		1,152(35.40)	
History of stroke disease, *n* (%)						
No	3,245(86.12)	*p* < 0.05	1,370(84.83)	*p* < 0.05	2,591(79.63)	*p* < 0.05
Yes	523(13.88)		245(15.17)		663(20.37)	
History of arthritis disease, *n* (%)						
No	1,648(43.74)	*p* > 0.05	707(43.78)	*p* > 0.05	1,412(43.39)	*p* > 0.05
Yes	2,120(56.26)		908(56.22)		1842(56.61)	
Outcome measurements						
Depressed, *n* (%)						
No	1,646(43.68)	*p* > 0.05	705(43.65)	*p* > 0.05	1,165(35.80)	p > 0.05
Yes	2,122(56.32)		910(56.35)		2089(64.20)	

**Figure 3:**
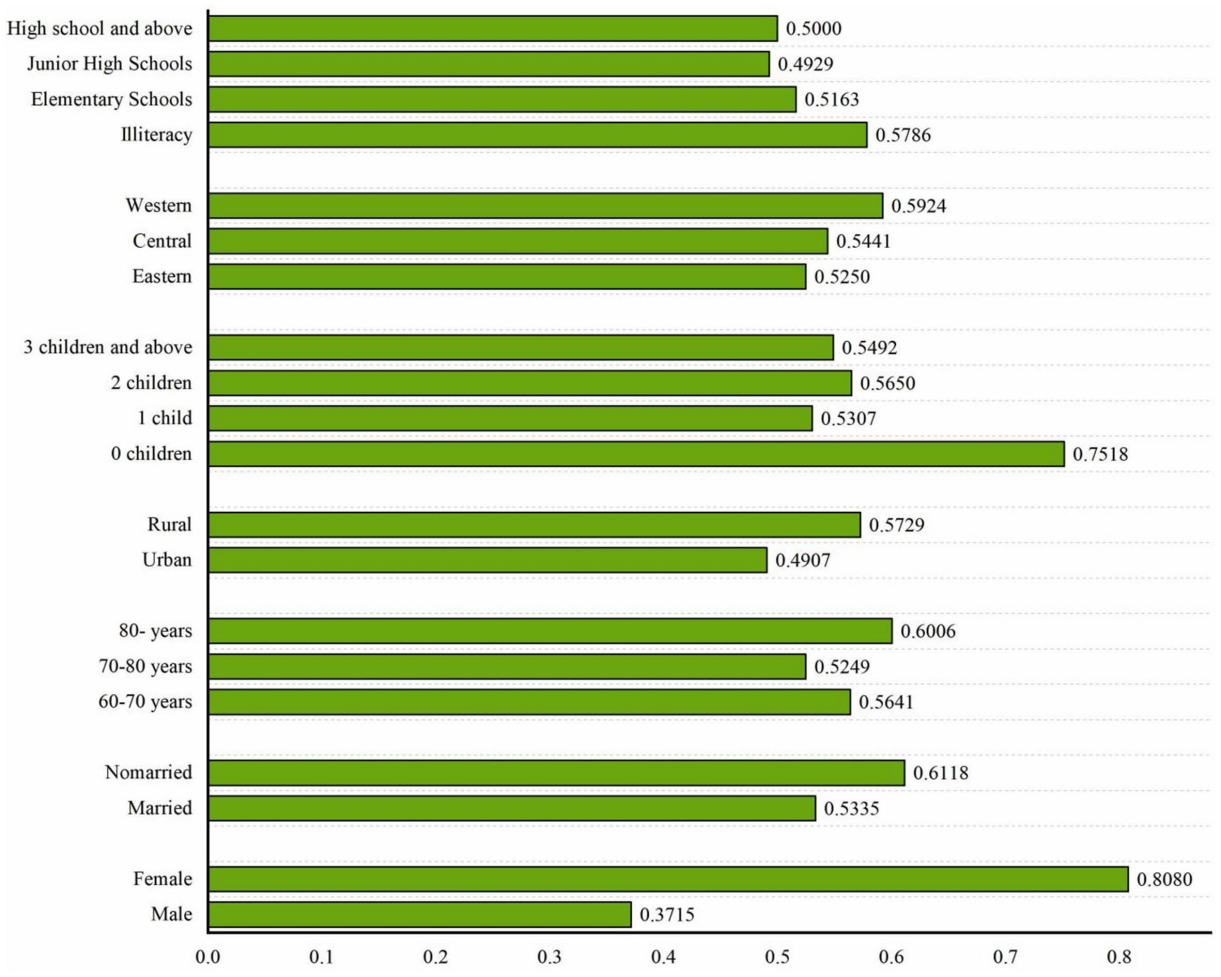
The demographic differences.

Figure 3 reveals the demographic differences in the prevalence of depression among disabled individuals. The gender distribution shows that the prevalence of depression in the female population (80.80%) is significantly higher than that in the male population (37.15%). Analysis of marital status shows that unmarried individuals have a higher risk of depression (61.18%) compared to married individuals (53.35%). In age stratification, the prevalence of depression in the elderly group aged 80 and above reached 60.06%, which was higher than that in the 70–80 age group (52.49%) and the 60–70 age group (56.41%). The regional distribution shows that the incidence rate in the western region (59.24%) and rural areas (57.29%) is significantly higher than that in the eastern region (52.50%) and urban areas (49.07%). Education level analysis shows that the illiterate population has the highest incidence of disease (57.86%), and there is a non-linear relationship between educational attainment and morbidity probability. The dimension of family support shows that the risk of depression in the childless group (75.18%) is significantly higher than that in the childbearing group (53.07–56.50%).

### 3.2 Model performance

This study systematically evaluated the performance of 10 ML algorithms in predicting depression risk among older adults with disability across training, testing, and external validation sets (as shown in Table 3). In terms of accuracy, RF (0.741), LightGBM (0.728), and HistGBM (0.713) demonstrated the highest performance in the testing sets. While LR and DT exhibited relatively stable performance between training and testing sets, their overall accuracy was the lowest (LR: 0.667; DT: 0.633). For AUC metrics, RF (0.797) achieved the strongest discriminative capacity, followed closely by LightGBM (0.785), XGBoost (0.781), and HistGBM (0.779). XGBoost, LightGBM, and HistGBM showed superior generalizability, whereas DT (0.636) performed the poorest. Regarding the F1-score, RF (0.762) exhibited the optimal balance between precision and recall, with LightGBM (0.749), CatBoost (0.741), and HistGBM (0.735) maintaining stable performance in the testing sets. For precision, RF (0.791), LightGBM (0.778), XGBoost (0.767), and HistGBM (0.723) achieved the highest positive predictive values and lowest false positive rates, significantly outperforming DT (0.691). In recall analysis, RF (0.735), HistGBM (0.723), and HistGBM (0.707) demonstrated the strongest ability to identify true positive cases, while DT (0.633) exhibited markedly higher missed-detection risks compared to ensemble methods.

**Table 3:** Performance of 10 ML algorithms on training and testing sets.

Model	Accuracy		AUC		F1-score		Precision		Recall	
	Train	Test	Train	Test	Train	Test	Train	Test	Train	Test
LR	0.702	0.667	0.781	0.723	0.689	0.685	0.721	0.734	0.661	0.642
HistGBM	0.782	0.713	0.864	0.779	0.778	0.735	0.793	0.766	0.763	0.707
MLP	0.805	0.698	0.882	0.761	0.797	0.718	0.830	0.758	0.767	0.682
XGBoost	0.801	0.713	0.882	0.781	0.797	0.735	0.813	0.767	0.782	0.705
Bagging	0.772	0.709	0.852	0.770	0.765	0.731	0.788	0.764	0.744	0.700
DT	0.651	0.633	0.653	0.636	0.648	0.661	0.654	0.691	0.642	0.633
LightGBM	0.803	0.728	0.883	0.785	0.799	0.749	0.816	0.778	0.782	0.723
RF	**0.822**	**0.741**	**0.901**	**0.797**	**0.817**	**0.762**	**0.840**	**0.791**	**0.796**	**0.735**
SVM	0.818	0.708	0.899	0.768	0.812	0.727	0.838	0.766	0.787	0.692
CatBoost	0.776	0.716	0.857	0.774	0.772	0.741	0.787	0.763	0.757	0.720

The highest value in each column is highlighted in bold. Final model selection prioritized generalizability across training, testing, and external validation sets, as detailed in the text.

**Figure 4:**
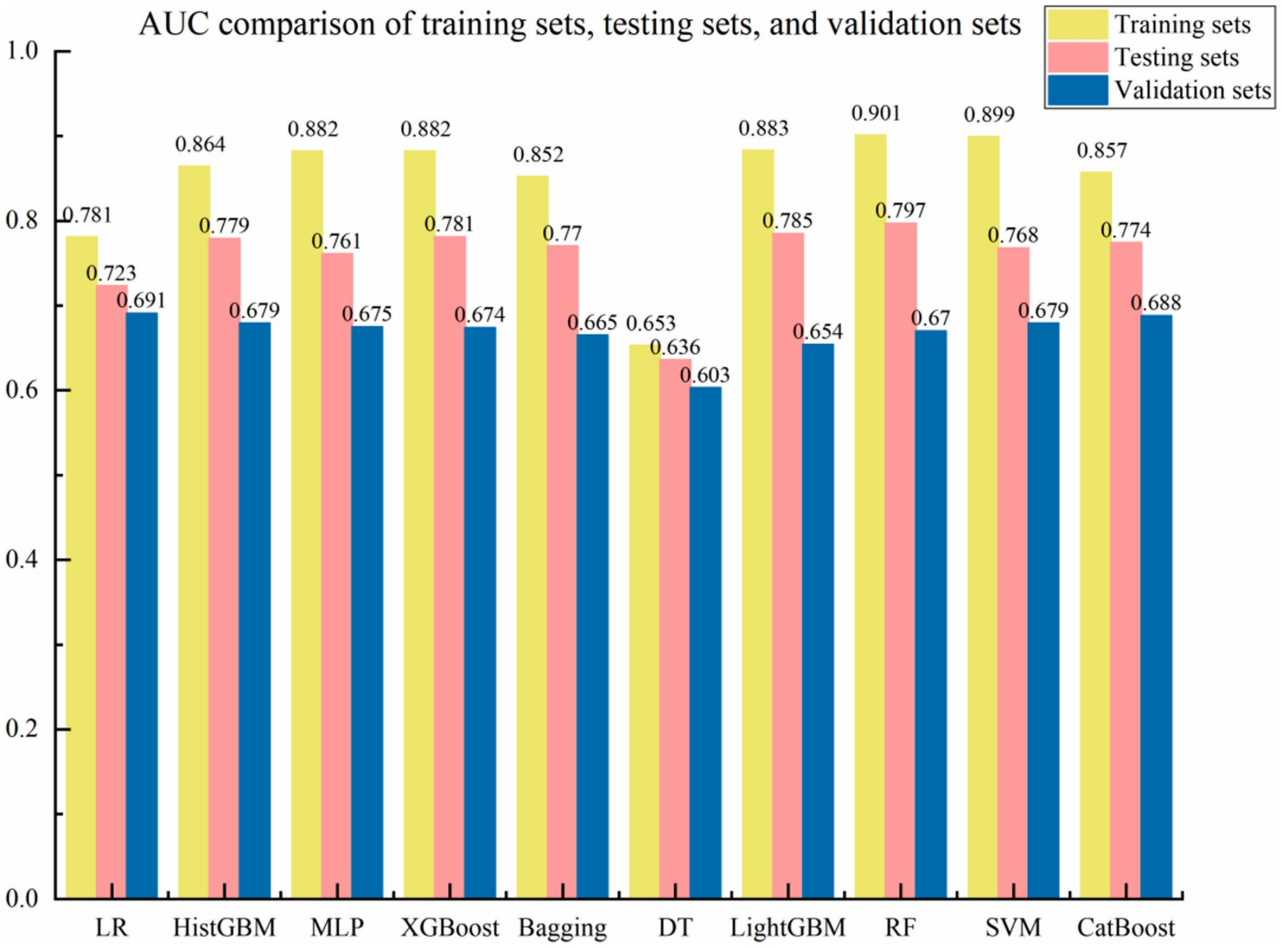
AUC comparison of training sets, testing sets, and validation sets.

Through comprehensive evaluation of 10 ML models, HistGBM was selected as the optimal model based on three key criteria: (1) superior performance on testing sets metrics (AUC = 0.779, F1-score = 0.735, accuracy = 0.713), (2) excellent generalizability demonstrated by a minimal training–testing AUC gap (8.5%), and (3) consistent performance across validation sets. HistGBM exhibited well-balanced predictive capabilities, showing above-average performance across all evaluation metrics without significant weaknesses in either precision (0.766) or recall (0.707), indicating robust discriminative power between positive and negative cases along with stable predictive performance. The model’s exceptional generalizability was particularly noteworthy, with only a 10% difference in AUC between the testing sets and validation sets, as shown in Figure 4, significantly outperforming other models and demonstrating strong robustness across different data distributions. Although RF achieved the highest individual metrics on the specific testing set used in Table 3, the substantial performance degradation observed on the external validation set raised concerns about its real-world applicability and stability. HistGBM, while having marginally lower peak testing set scores than RF, offered the best overall package of strong predictive performance, minimal overfitting (small train-test gap), and exceptional stability across the independent validation set. This superior generalizability was the primary reason for selecting HistGBM as the optimal model for potential clinical application, where reliability across diverse data sources is paramount.

**Figure 5:**
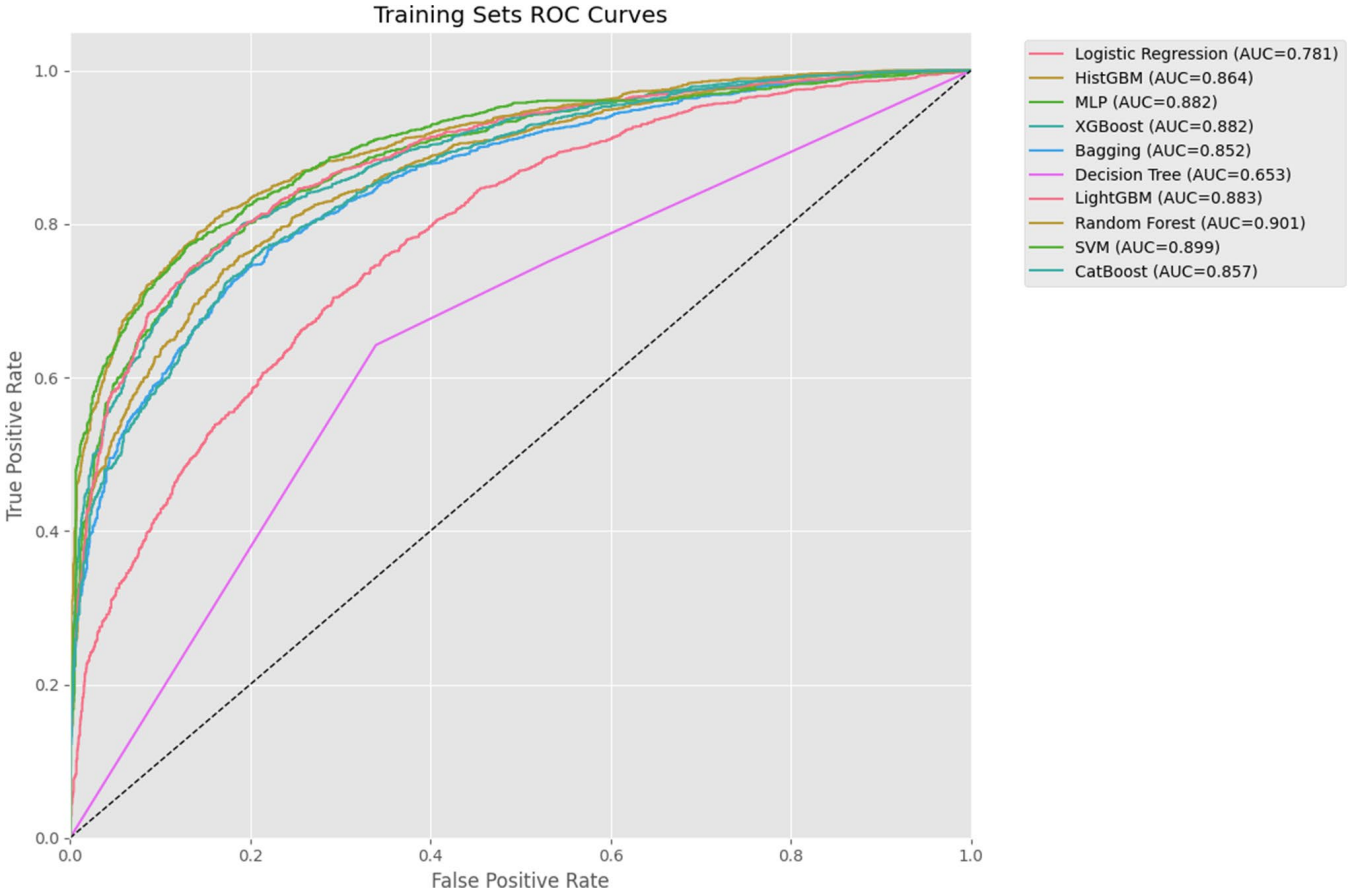
Training sets ROC curves.

**Figure 6:**
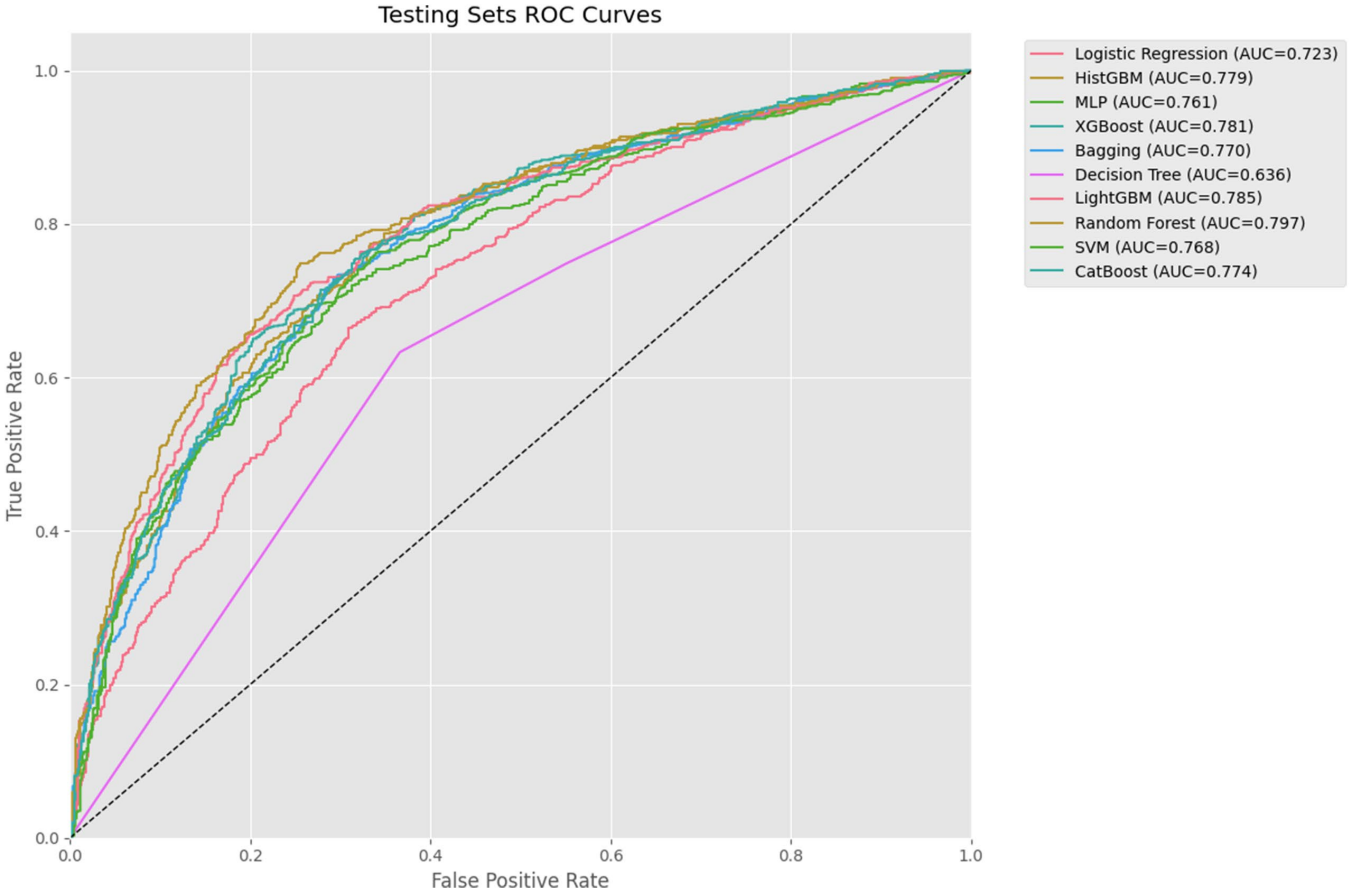
Testing sets the ROC curves.

**Figure 7:**
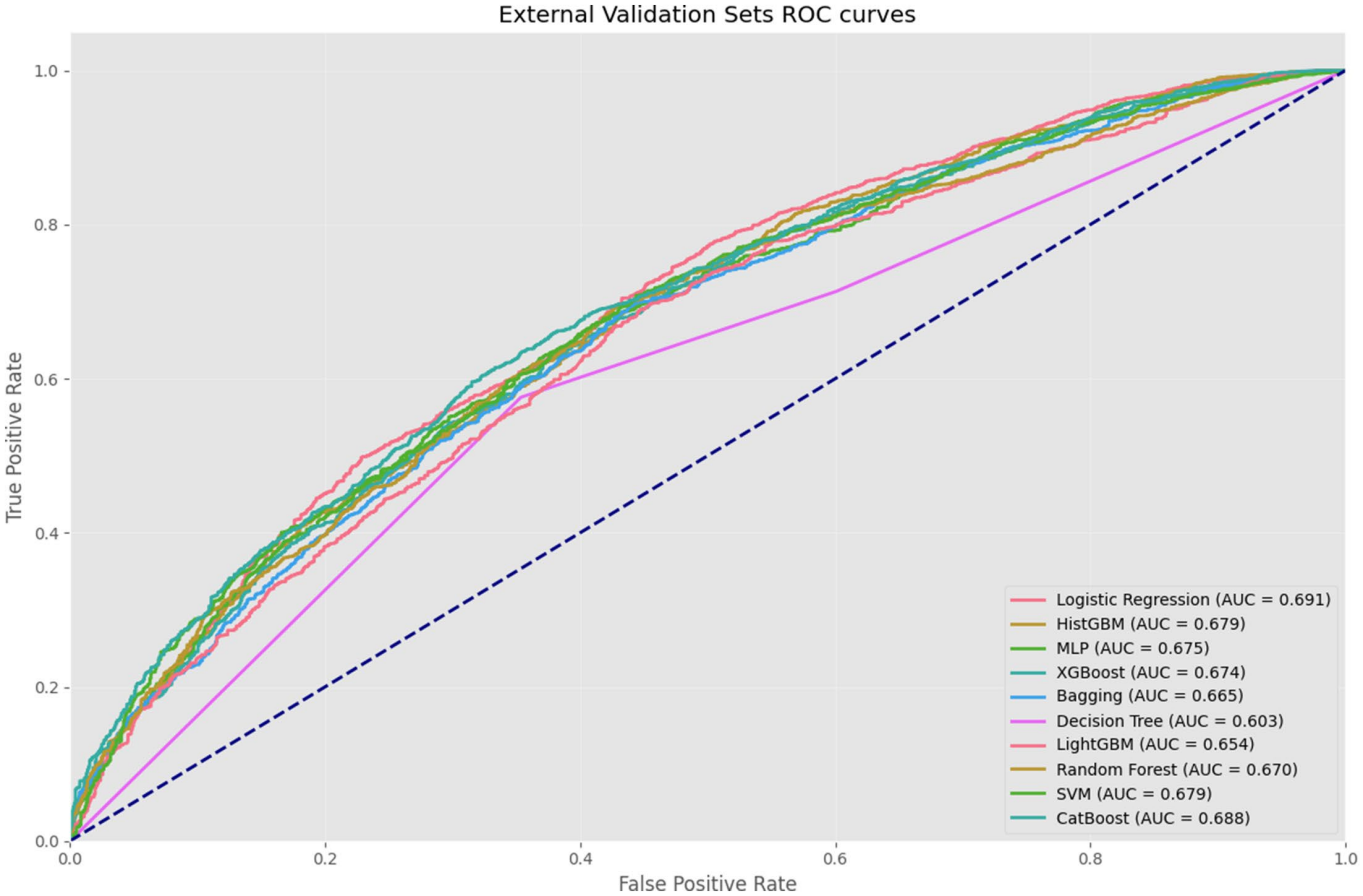
External validation sets ROC curves.

XGBoost demonstrated strong performance on testing sets metrics (AUC = 0.781, F1-score = 0.735, accuracy = 0.713), though it exhibited a relatively large training–testing AUC gap (10.1%) compared to LR, HistGBM, MLP, and CatBoost. However, its validation set’s AUC showed a 10.7% difference from testing sets’ performance (Figures 5–7), suggesting reasonable stability across data partitions and potential suitability for resource-constrained scenarios. RF achieved excellent testing set results (AUC = 0.797, F1-score = 0.762, accuracy = 0.741), but displayed concerning generalization issues with substantial training–testing and testing-validation AUC differences, indicating potential overfitting to training data noise or specific patterns. LightGBM ranked second in testing sets AUC (0.785) with stable validation performance (0.654). However, the difference between the testing set AUC and the validation set is too large (13.1%). CatBoost performed comparably to top models in testing set metrics (AUC = 0.774, F1-score = 0.741) with excellent categorical feature handling, though its relatively lower F1-score (0.741) and recall (0.720) scores suggested weaker minority class identification, limiting its utility for imbalanced datasets. Among the remaining models, SVM exhibited severe overfitting, while MLP and DT significantly underperformed ensemble methods in both AUC and F1-score metrics.

### 3.3 Model explanation

**Figure 8:**
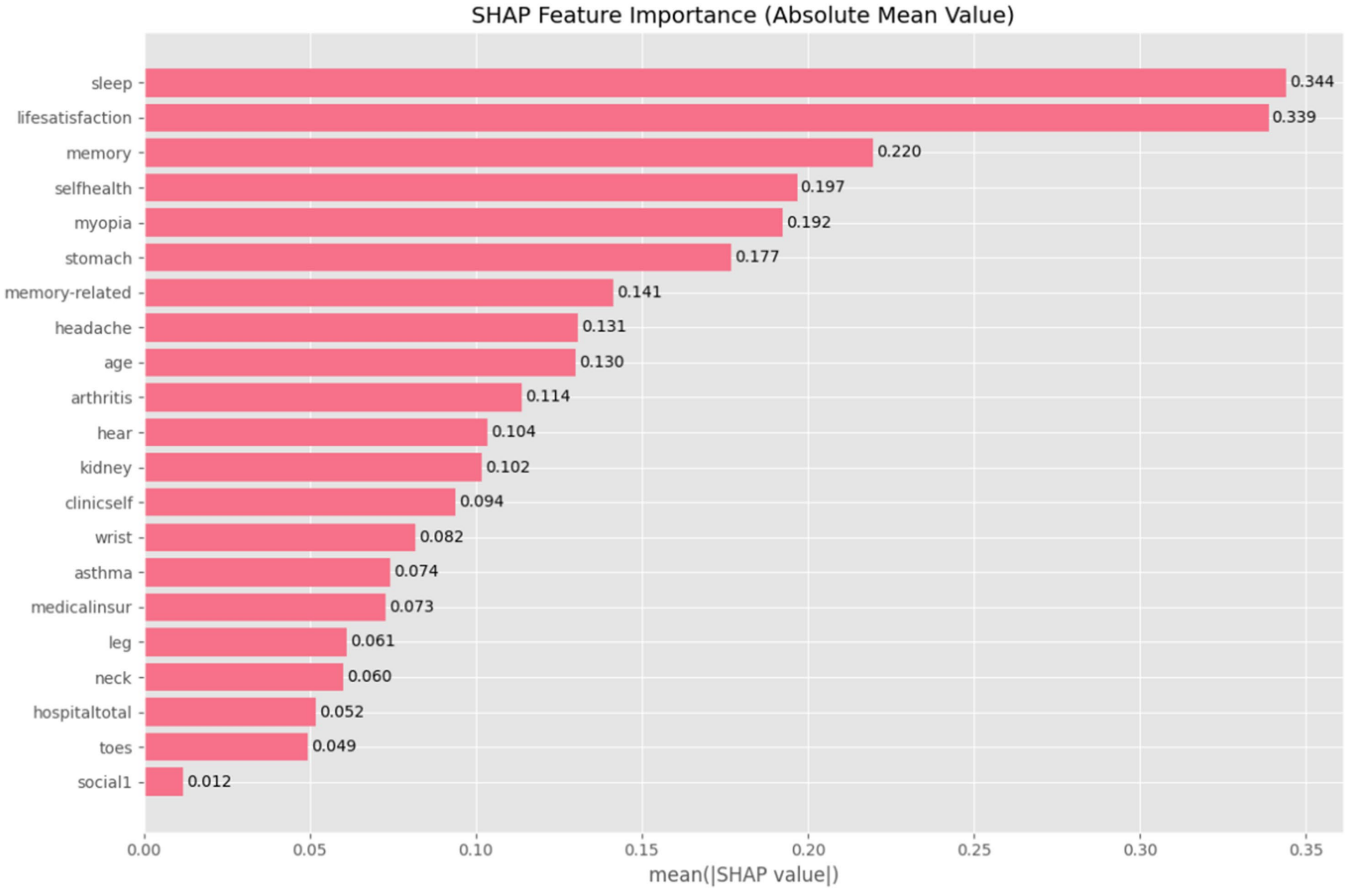
SHAP feature importance.

The SHAP values quantify the absolute average impact of each feature on model predictions across all possible feature combinations, revealing their global importance. As shown in Figure 8, the SHAP analysis of the HistGBM model demonstrated significant variability in feature contributions. Sleep time (mean SHAP = 0.344), life satisfaction (0.339), episodic memory (0.220), and self-rated health (0.197) emerged as the top four predictive features, indicating that health behaviors, subjective perceptions, and cognitive function were the core drivers of model predictions. The high contribution of sleep time likely reflects its well-established associations with chronic diseases, metabolic disorders, and cognitive decline. Life satisfaction and self-rated health, as subjective health indicators, capture the interplay between psychosocial factors and physiological health. Episodic memory directly influences prediction through cognitive and sensory pathways. Moderate contributions were observed for features such as stomach diseases, observing the situation up close, and memory-related disorders. While self-reported pain in the head, wrist, leg, toes, neck, and mental health conditions showed limited predictive importance, suggesting either weak signals or sparse data distributions. These results validate the model’s multidimensional feature selection approach and provide actionable insights for intervention prioritization. Health management strategies targeting high-contribution features could enhance the model’s real-world utility. Additionally, domain knowledge should guide the evaluation of low-contribution features to optimize the balance between model complexity and interpretability.

**Figure 9:**
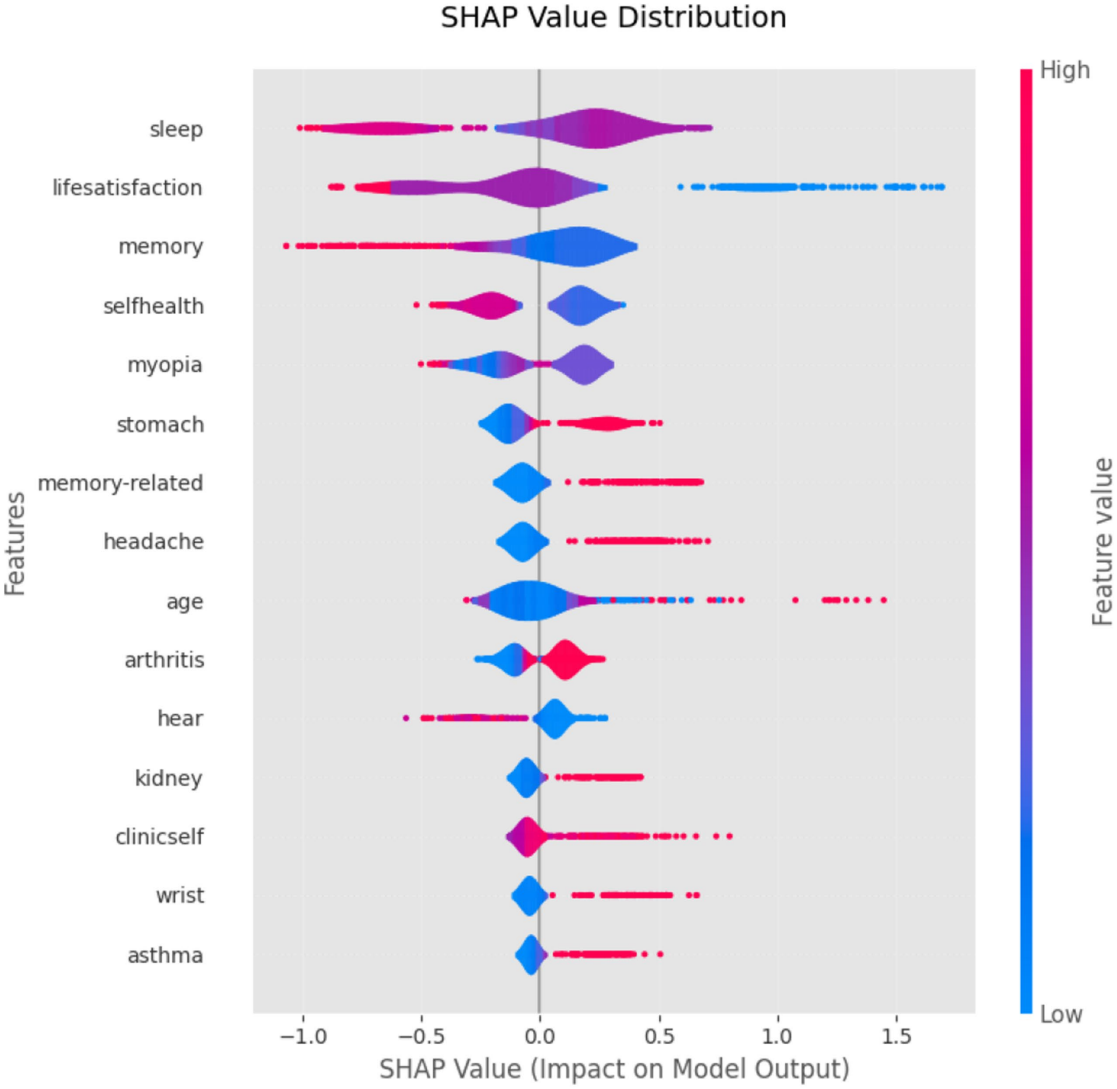
SHAP value distribution.

Figure 9 presents the SHAP value distributions, revealing the heterogeneous directional effects and magnitudes of various features on depression probability predictions among older adults with disability. The x-axis (SHAP value) indicates each feature’s influence on model output, where positive values increase and negative values decrease predicted risk. The color gradient (red means high feature value, blue means low feature value) demonstrates that: (1) higher values of sleep time, life satisfaction, and self-rated health (red clusters with negative SHAP) were strongly protective against depression, consistent with established epidemiological mechanisms; (2) better episodic memory performance (blue with positive SHAP) correlated with reduced depression risk, potentially through preserved cognitive resilience; (3) stomach diseases (red with positive SHAP) elevated risk through chronic somatic burden and psychological stress pathways; and (4) bodily pain (head, wrist, leg, toes, neck; red with positive SHAP) increased depression vulnerability in this population. These findings highlight the central role of health behaviors and psychosocial factors in depression comorbidity risk while identifying specific physiological pain features as contributory predictors.

## 4 Discussion

This study identified significant associations between depressive risk among disabled older adults and demographic characteristics, health status, and social support factors. The external validation cohort’s higher median age than training sets and elevated depression risk in advanced age align with existing literature (Zhao et al., 2012; Gao et al., 2023), potentially mediated by cognitive decline and reduced social roles. Consistent with Girgus et al. (2017), females demonstrated significantly higher risk than males, possibly due to gender-specific social expectations, somatic symptom expression patterns, and help-seeking behaviors (Handing et al., 2022). The elevated risk among unmarried individuals supports the marital support hypothesis, where spousal emotional and economic support may serve as protective factors (Soulsby and Bennett, 2015), corroborating Zhai et al. (2024). Notably, the higher prevalence in rural western regions reflects China’s geographic disparities in healthcare resource allocation, echoing Fan et al. (2022) on primary mental health service accessibility. Higher education levels were protective, consistent with prior studies (Li et al., 2022; Kondirolli and Sunder, 2022), likely through multiple pathways: enhanced cognitive capacity, improved socioeconomic resources, greater mental health awareness, and healthier behaviors. The elevated risk among childless individuals suggests family support network deficiencies may exacerbate disability-related stress, particularly relevant in East Asian familial care traditions (Feng, 2018), though some studies report no direct mental health impact of childlessness (Koropeckyj-Cox, 1998; Quashie et al., 2021).

This study systematically evaluated 10 ML models (LR, SVM, XGBoost, LightGBM, CatBoost, RF, Bagging, HistGBM, MLP, DT) for predicting depressive risk among disabled older adults, demonstrating the superior performance of ensemble methods over traditional approaches. The HistGBM algorithm achieved optimal predictive accuracy, with AUC values of 0.779 (testing sets), aligning with current trends in medical prediction research. While Busi and Stephen (2023) similarly compared extreme gradient boosting methods for early kidney disease diagnosis, their study did not examine the generalization enhancement effects of histogram optimization. Lee et al. (2022) likewise identified extreme gradient boosting as the top performer for chronic disease prediction (AUC ≥ 0.80). HistGBM’s minimal AUC divergence between validation and testing sets (10%) confirms that histogram binning effectively mitigates overfitting caused by high-dimensional sparse features characteristic of healthcare data.

Notably, while RF achieved the highest testing sets AUC (0.797), its validation performance showed significant degradation (ΔAUC = 12.7%), contrasting sharply with its training sets performance (AUC = 10.4%). This suggests that RF’s majority voting mechanism may amplify localized features in training data when strong collinearity or noise exists in the feature space (Anil and Singh, 2023). In comparison, HistGBM maintained tighter training–testing consistency (8.5% AUC difference), outperforming both XGBoost (10.1%) and LightGBM (9.8%), indicating its superior suitability for handling elderly health data with measurement errors. Regarding class imbalance handling, XGBoost demonstrated significantly lower recall (0.705) than HistGBM (0.707), consistent with findings by Baba and Bunji (2023). HistGBM’s adaptive histogram partitioning mechanism balanced class weights while maintaining high precision (0.766), yielding a 5% F1-score improvement over LR (0.685). This enhancement likely stems from our feature engineering strategy that deeply explored disability-related psychosocial variables. These findings provide new empirical evidence for model selection in medical ML applications.

The SHAP interpretability framework revealed multidimensional drivers of depressive risk prediction among disabled older adults. Sleep time emerged as the primary predictor (SHAP = 0.344), demonstrating significantly greater contribution than reported in the Song et al. (2022) study (SHAP = 0.133). This enhanced predictive importance may reflect disability-associated sleep fragmentation, potentially activating inflammatory pathways and amplifying pathological effects. Notably, life satisfaction (SHAP = 0.339) and self-rated health (SHAP = 0.197) demonstrated greater predictive influence than conventional biomedical indicators, establishing psychosocial factors as pivotal determinants in depression comorbidity mechanisms among individuals with disability (Santos et al., 2013). Within cognitive domains, episodic memory (SHAP = 0.220) showed higher predictive contribution than observing the situation up close (SHAP = 0.192). Conversely, the relatively low importance of somatic pain features suggests the need to reevaluate clinical assessment priorities for pain screening in this population.

This study achieves a breakthrough by integrating longitudinal design, ML techniques, and multidimensional feature engineering. First, compared to cross-sectional designs, our multi-wave feature construction quantitatively captures the cumulative effects of factors such as sleep disturbance (SHAP value = 0.344). Second, in contrast to conventional statistical models, the HistGBM algorithm significantly enhances generalizability through histogram optimization (training–testing AUC gap: 8.5%). Third, the predictive contribution of subjective perception indicators (life satisfaction SHAP value = 0.339) surpasses that of traditional physiological measures, validating our novel finding that psychosocial features dominate depression risk prediction. These methodological innovations collectively advance the field by providing a more robust, dynamic, and interpretable framework for risk stratification.

While this study provides valuable insights into depressive disorder risk stratification in functionally impaired geriatric populations, several limitations should be acknowledged. First, the reliance on the CHARLS self-reported measures may lead to an underestimation of both disability severity and depressive symptoms. Second, the exclusion of biomarkers limits the model’s ability to differentiate depression subtypes. Third, the absence of real-time dynamic health monitoring data potentially reduces the predictive value of temporal features. Future research should incorporate wearable device data and multi-omics approaches to develop dynamic prediction systems, complemented by cross-cohort validation to enhance generalizability. Fourth, our disability definition focused exclusively on BADL/IADL limitations. While this is consistent with geriatric assessment standards, it may not capture populations with pure cognitive or sensory disabilities. However, this standardized approach minimized cohort heterogeneity, facilitating model training on uniformly defined functional impairments. Future studies should validate these findings in other disability subtypes.

## 5 Conclusion

This study constructed a clinically generalizable prediction model for depressive risk among disabled older adults by integrating longitudinal data from multiple CHARLS waves. Our three-stage serial consensus approach feature selection system identified 21 robust predictors spanning physiological function, social support, and health behaviors, overcoming limitations of traditional linear modeling approaches. The HistGBM algorithm demonstrated optimal predictive stability through its histogram binning technique and adaptive learning mechanism. SHAP interpretability analysis revealed that health behavior (sleep time) and subjective perception indicators (life satisfaction, self-rated health) contributed significantly more to predictions than biomedical features, underscoring the central importance of psychosocial interventions in depression prevention for this population. The study identified significantly elevated depression risks among specific demographic subgroups with disability, including individuals residing in western rural regions, elderly females, those with limited educational attainment, and childless older adults. These findings highlight the urgent need for community-based mental health service networks and family support policies. These results provide an evidence base for preventing psychological disorders and implementing mental health interventions among the aging population with disability.

## Data Availability

The original contributions presented in the study are included in the article/Supplementary material, further inquiries can be directed to the corresponding author, Ayitijiang·Halili.

## References

[ref1] Abdoli N. Salari N. Darvishi N. Jafarpour S. Solaymani M. Mohammadi M.. (2022). The global prevalence of major depressive disorder (MDD) among the elderly: a systematic review and meta-analysis. Neurosci. Biobehav. Rev. 132, 1067–1073. doi: 10.1016/j.neubiorev.2021.10.041, PMID: 34742925

[ref2] Ai F. Li E. Ji Q. Zhang H. (2024). Construction of a machine learning-based risk prediction model for depression in middle-aged and elderly hypertensive people in China: a longitudinal study. Front. Psych. 15:96. doi: 10.3389/fpsyt.2024.1398596, PMID: 38764471 PMC11099225

[ref3] Anil A. K. P. Singh U. K. (2023). An optimal solution to the overfitting and underfitting problem of healthcare machine learning models. J. Syst. Eng. Inf. Technol. 2, 77–84. doi: 10.29207/joseit.v2i2.5460

[ref4] Asdaq S. M. B. Alshehri S. Alajlan S. A. Almutiri A. A. Alanazi A. K. R. (2024). Depression in persons with disabilities: a scoping review. Front. Public Health 12:78. doi: 10.3389/fpubh.2024.1383078 PMC11110534 38779421

[ref5] Baba A. Bunji K. (2023). Prediction of mental health problem using annual student health survey: machine learning approach. JMIR Ment. Health 10:e42420. doi: 10.2196/42420, PMID: 37163323 PMC10209795

[ref6] Busi R. A. Stephen M. J. (2023). Effective classification of chronic kidney disease using extreme gradient boosting algorithm. Int. J. Softw. Innov. 11, 1–18. doi: 10.4018/IJSI.315732

[ref7] Çağan Ö. Ünsal A. (2014). Depression and loneliness in disabled adults. Procedia. Soc. Behav. Sci. 114, 754–760. doi: 10.1016/j.sbspro.2013.12.780

[ref8] Chen H. Mui A. C. (2014). Factorial validity of the Center for Epidemiologic Studies Depression Scale short form in older population in China. Int. Psychogeriatr. 26, 49–57. doi: 10.1017/S1041610213001701, PMID: 24125553

[ref9] Cheng S.-T. Chan A. C. M. (2005). The Center for Epidemiologic Studies Depression Scale in older Chinese: thresholds for long and short forms. Int. J. Geriatr. Psychiatry 20, 465–470. doi: 10.1002/gps.1314, PMID: 15852439

[ref10] Chu J. Li Y. Wang X. Xu Q. Xu Z. (2025). Development of a longitudinal model for disability prediction in older adults in China: analysis of CHARLS data (2015-2020). JMIR Aging 8:e66723. doi: 10.2196/66723, PMID: 40247464 PMC12021300

[ref11] Fan H. Li S. Guo X. Chen M. Zhang H. Chen Y. (2025). Development and validation of a machine learning-based diagnostic model for Parkinson’s disease in community-dwelling populations: evidence from the China health and retirement longitudinal study (CHARLS). Parkinsonism Relat. Disord. 130:107182. doi: 10.1016/j.parkreldis.2024.107182, PMID: 39522387

[ref12] Fan X. Zhang W. Guo Y. Cai J. Xie B. (2022). Equity assessment of the distribution of mental health beds in China: based on longitudinal data from 2011 to 2020. BMC Health Serv. Res. 22:1453. doi: 10.1186/s12913-022-08658-z, PMID: 36451145 PMC9709752

[ref13] Feng Z. (2018). Childlessness and vulnerability of older people in China. Age Ageing 47, 275–281. doi: 10.1093/ageing/afx137, PMID: 29096004 PMC6016684

[ref14] Feng Z. Li Q. Zhou L. Chen Z. Yin W. (2021). The relationship between depressive symptoms and activity of daily living disability among the elderly: results from the China health and retirement longitudinal study (CHARLS). Public Health 198, 75–81. doi: 10.1016/j.puhe.2021.06.023, PMID: 34365109

[ref15] Gao X. Geng T. Jiang M. Huang N. Zheng Y. Belsky D. W.. (2023). Accelerated biological aging and risk of depression and anxiety: evidence from 424,299 UK biobank participants. Nat. Commun. 14:2277. doi: 10.1038/s41467-023-38013-7, PMID: 37080981 PMC10119095

[ref16] Girgus J. S. Yang K. Ferri C. V. (2017). The gender difference in depression: are elderly women at greater risk for depression than elderly men? Geriatrics 2:35. doi: 10.3390/geriatrics2040035, PMID: 31011045 PMC6371140

[ref17] Han Y. Wang S. (2023). Disability risk prediction model based on machine learning among Chinese healthy older adults: results from the China health and retirement longitudinal study. Front. Public Health 11:595. doi: 10.3389/fpubh.2023.1271595, PMID: 38026309 PMC10665855

[ref18] Handing E. P. Strobl C. Jiao Y. Feliciano L. Aichele S. (2022). Predictors of depression among middle-aged and older men and women in Europe: a machine learning approach. Lancet Reg. Health 18:391. doi: 10.1016/j.lanepe.2022.100391, PMID: 35519235 PMC9065918

[ref19] Hong S. Lu B. Wang S. Jiang Y. (2025). Comparison of logistic regression and machine learning methods for predicting depression risks among disabled elderly individuals: results from the China health and retirement longitudinal study. BMC Psychiatry 25:128. doi: 10.1186/s12888-025-06577-x, PMID: 39953491 PMC11829540

[ref20] Huang Q. Jiang Z. Shi B. Meng J. Shu L. Hu F.. (2025). Characterisation of cardiovascular disease (CVD) incidence and machine learning risk prediction in middle-aged and elderly populations: data from the China health and retirement longitudinal study (CHARLS). BMC Public Health 25:518. doi: 10.1186/s12889-025-21609-7, PMID: 39920658 PMC11806717

[ref21] Katz S. Ford A. B. Moskowitz R. W. Jackson B. A. Jaffe M. W. (1963). Studies of illness in the aged. The index of ADL: a standardized measure of biological and psychosocial function. JAMA 185, 914–919. doi: 10.1001/jama.1963.03060120024016, PMID: 14044222

[ref22] Kondirolli F. Sunder N. (2022). Mental health effects of education. Health Econ. 31, 22–39. doi: 10.1002/hec.4565, PMID: 35797349 PMC9796491

[ref23] Koropeckyj-Cox T. (1998). Loneliness and depression in middle and old age: are the childless more vulnerable? J. Gerontol. B Psychol. Sci. Soc. Sci. 53, S303–S312. doi: 10.1093/geronb/53b.6.s303, PMID: 9826972

[ref24] Kupferberg A. Bicks L. Hasler G. (2016). Social functioning in major depressive disorder. Neurosci. Biobehav. Rev. 69, 313–332. doi: 10.1016/j.neubiorev.2016.07.002, PMID: 27395342

[ref25] Lawton M. P. Brody E. M. (1969). Assessment of older people: self-maintaining and instrumental activities of daily living. Gerontologist 9, 179–186. doi: 10.1093/geront/9.3_Part_1.179, PMID: 5349366

[ref26] Lee C. Jo B. Woo H. Im Y. Park R. W. Park C. (2022). Chronic disease prediction using the common data model: development study. JMIR AI 1:e41030. doi: 10.2196/41030, PMID: 38875545 PMC11041444

[ref27] Li L. Sun W. Luo J. Huang H. (2022). Associations between education levels and prevalence of depressive symptoms: NHANES (2005–2018). J. Affect. Disord. 301, 360–367. doi: 10.1016/j.jad.2022.01.010, PMID: 34990632

[ref28] McGillivray J. A. McCabe M. P. (2007). Early detection of depression and associated risk factors in adults with mild/moderate intellectual disability. Res. Dev. Disabil. 28, 59–70. doi: 10.1016/j.ridd.2005.11.001, PMID: 16517122

[ref29] Mu T.-Y. Xu R.-X. Xu J.-Y. Dong D. Zhou Z.-N. Dai J.-N.. (2022). Association between self-care disability and depressive symptoms among middle-aged and elderly Chinese people. PLoS One 17:e0266950. doi: 10.1371/journal.pone.0266950, PMID: 35404987 PMC9000112

[ref30] Musliner K. L. Munk-Olsen T. Eaton W. W. Zandi P. P. (2016). Heterogeneity in long-term trajectories of depressive symptoms: patterns, predictors and outcomes. J. Affect. Disord. 192, 199–211. doi: 10.1016/j.jad.2015.12.030, PMID: 26745437 PMC4761648

[ref31] Ni Z. Zhu X. Tian K. Chen Q. Yang Y. Xie S. (2024). Depressive symptoms of older adults with chronic diseases: the mediating roles of activities of daily living and economic burden of diseases. Front. Psychol. 15:1387677. doi: 10.3389/fpsyg.2024.1387677, PMID: 39015326 PMC11249773

[ref32] Nickson D. Meyer C. Walasek L. Toro C. (2023). Prediction and diagnosis of depression using machine learning with electronic health records data: a systematic review. BMC Med. Inform. Decis. Mak. 23:271. doi: 10.1186/s12911-023-02341-x, PMID: 38012655 PMC10680172

[ref33] Quashie N. T. Arpino B. Antczak R. Mair C. A. (2021). Childlessness and health among older adults: variation across five outcomes and 20 countries. J. Gerontol. B Psychol. Sci. Soc. Sci. 76, 348–359. doi: 10.1093/geronb/gbz153, PMID: 31768550

[ref34] Santos V. Paes F. Pereira V. Arias-Carrión O. Silva A. C. Carta M. G.. (2013). The role of positive emotion and contributions of positive psychology in depression treatment: systematic review. Clin. Pract. Epidemiol. Ment. Health 9, 221–237. doi: 10.2174/1745017901309010221, PMID: 24358052 PMC3866689

[ref35] Song S. DeMeo N. N. Almeida D. M. Majd M. Engeland C. G. Graham-Engeland J. E. (2022). The longitudinal connection between depressive symptoms and inflammation: mediation by sleep quality. PLoS One 17:e0269033. doi: 10.1371/journal.pone.0269033, PMID: 35617264 PMC9135207

[ref36] Song Y. Qian L. Sui J. Greiner R. Li X. Greenshaw A. J.. (2023). Prediction of depression onset risk among middle-aged and elderly adults using machine learning and Canadian longitudinal study on aging cohort. J. Affect. Disord. 339, 52–57. doi: 10.1016/j.jad.2023.06.031, PMID: 37380110

[ref37] Soulsby L. K. Bennett K. M. (2015). Marriage and psychological wellbeing: the role of social support. Psychology 6, 1349–1359. doi: 10.4236/psych.2015.611132

[ref38] Theerthagiri P. (2022). Predictive analysis of cardiovascular disease using gradient boosting based learning and recursive feature elimination technique. Intell. Syst. Appl. 16:200121. doi: 10.1016/j.iswa.2022.200121

[ref39] Tian F. Yang H. Pan J. (2022). Association between functional disability and long-term trajectories of depressive symptoms: evidence from the China health and retirement longitudinal study. J. Affect. Disord. 310, 10–16. doi: 10.1016/j.jad.2022.05.001, PMID: 35525506

[ref40] Turner R. J. Noh S. (1988). Physical disability and depression: a longitudinal analysis. J. Health Soc. Behav. 29, 23–37. doi: 10.2307/2137178, PMID: 2966841

[ref41] Vu T. Dawadi R. Yamamoto M. Tay J. T. Watanabe N. Kuriya Y.. (2025). Prediction of depressive disorder using machine learning approaches: findings from the NHANES. BMC Med. Inform. Decis. Mak. 25:83. doi: 10.1186/s12911-025-02903-1, PMID: 39962516 PMC11834192

[ref42] Wang J. Luo N. Sun Y. Bai R. Li X. Liu L.. (2023). Exploring the reciprocal relationship between activities of daily living disability and depressive symptoms among middle-aged and older Chinese people: a four-wave, cross-lagged model. BMC Public Health 23:1180. doi: 10.1186/s12889-023-16100-0, PMID: 37337186 PMC10280867

[ref43] Wang M. Su W. Chen H. Li H. (2023). Depressive symptoms and risk of incident cardiometabolic multimorbidity in community-dwelling older adults: the China health and retirement longitudinal study. J. Affect. Disord. 335, 75–82. doi: 10.1016/j.jad.2023.04.048, PMID: 37075824

[ref44] Xin Y. Ren X. (2022). Predicting depression among rural and urban disabled elderly in China using a random forest classifier. BMC Psychiatry 22:118. doi: 10.1186/s12888-022-03742-4, PMID: 35168579 PMC8845343

[ref45] Yan Y. Du Y. Li X. Ping W. Chang Y. (2023). Physical function, ADL, and depressive symptoms in Chinese elderly: evidence from the CHARLS. Front. Public Health 11:1017689. doi: 10.3389/fpubh.2023.1017689, PMID: 36923048 PMC10010774

[ref46] Yang T. Ying Y. (2022). AUC maximization in the era of big data and AI: a survey. ACM Comput. Surv. 55, 1–37. doi: 10.1145/3554729

[ref47] Zhai X. Tong H. H. Y. Lam C. K. Xing A. Sha Y. Luo G.. (2024). Association and causal mediation between marital status and depression in seven countries. Nat. Hum. Behav. 8, 2392–2405. doi: 10.1038/s41562-024-02033-0, PMID: 39496771

[ref48] Zhang X. Xue M. Zhang Z. Gao Z. Li C. Wu J.. (2024). Impact of social, familial and personal factors on depressive symptoms in middle-aged and older adults from the national CHARLS cohort. BMC Public Health 24:2669. doi: 10.1186/s12889-024-20159-8, PMID: 39350109 PMC11440718

[ref49] Zhao K.-X. Huang C.-Q. Xiao Q. Gao Y. Liu Q.-X. Wang Z.-R.. (2012). Age and risk for depression among the elderly: a meta-analysis of the published literature. CNS Spectr. 17, 142–154. doi: 10.1017/S1092852912000533, PMID: 22892113

[ref50] Zhao X. Wang Y. Li J. Liu W. Yang Y. Qiao Y.. (2025). A machine-learning-derived online prediction model for depression risk in COPD patients: a retrospective cohort study from CHARLS. J. Affect. Disord. 377, 284–293. doi: 10.1016/j.jad.2025.02.063, PMID: 39988142

[ref51] Zheng Y. Zhang T. Yang S. Wang F. Zhang L. Liu Y. (2024). Using machine learning to predict the probability of incident 2-year depression in older adults with chronic diseases: a retrospective cohort study. BMC Psychiatry 24:870. doi: 10.1186/s12888-024-06299-6, PMID: 39623372 PMC11610371

[ref52] Zhou L. Wang W. Ma X. (2024). The bidirectional association between the disability in activities of daily living and depression: a longitudinal study in Chinese middle-aged and older adults. BMC Public Health 24:1884. doi: 10.1186/s12889-024-19421-w, PMID: 39010036 PMC11247890

[ref53] Zhu G. Song Y. Lu Z. Yi Q. Xu R. Xie Y.. (2025). Machine learning models for predicting metabolic dysfunction-associated steatotic liver disease prevalence using basic demographic and clinical characteristics. J. Transl. Med. 23:381. doi: 10.1186/s12967-025-06387-5, PMID: 40155991 PMC11951774

[ref54] Zhu X. Wang Y. Luo Y. Ding R. Shi Z. He P. (2024). Bidirectional, longitudinal associations between depressive symptoms and IADL/ADL disability in older adults in China: a national cohort study. BMC Geriatr. 24:659. doi: 10.1186/s12877-024-05248-y, PMID: 39107705 PMC11301930

