# Correction: Predicting the risk of depression in older adults with disability using machine learning: an analysis based on CHARLS data

**DOI:** 10.3389/frai.2025.1659362

**Published:** 2025-07-23

**Authors:** Tongtong Jin, Ayitijiang· Halili

**Affiliations:** ^1^School of Law, Shanxi University of Finance and Economics, Taiyuan, China; ^2^College of Public Management (Law), Xinjiang Agricultural University, Urumqi, China

**Keywords:** disabled older adults, depression, risk prediction, machine learning, CHARLS, mental health LR, HistGBM, MLP

Author Ayitijiang·Halili was erroneously written as Ayitijiang Halili.

Affiliation ^1^School of Law, Shanxi University of Finance and Economics, Taiyuan, China was erroneously written as: ^1^College of Public Management (Law), Shanxi University of Finance and Economics, Taiyuan, China.

Affiliation ^2^College of Public Management (Law), Xinjiang Agricultural University, Urumqi, China was erroneously written as ^2^School of Law, Xinjiang Agricultural University, Urumqi, China.

The original version of this article has been updated.

