# Supplementary material for: Predicting the risk of depression in older adults with disability using machine learning: an analysis based on CHARLS data
Source: Front Artif Intell. 2025 Jul 2;8:1624171. doi: 10.3389/frai.2025.1624171 (PMC12263909; doi:10.3389/frai.2025.1624171)
Supplement: Supplementary file 1 [file Table_1.docx]

Supplementary Material

# Supplementary Tables

**Supplementary Table 1.** Variable description and assignment.

| Variables | Variable assignment |
| --- | --- |
| Outcome variables | |
| Depression | Assessed via a 10-item scale (score range: 0-30) evaluating the following domains over the past week: bothered by things, difficulty concentrating, depressed mood, everything feels like a struggle, hopefulness about the future, fearful, sleep was restless, happy, lonely, unable to continue living |
| Disability | Activities of Daily Living (ADL) |
| Basic Activities of Daily Living (BADL) | Composite score (0-6) encompassing dressing, bathing, eating, getting out of bed, using the toilet, controlling urination and defecation |
| Instrumental Activities of Daily Living (IADL) | Composite score (0-5) evaluating doing chores, preparing hot meals, shopping, managing money, taking medications |
| Demographic backgrounds | |
| Gender | 1=Male, 0=Female |
| Age |  |
| Registered residence | 1=Rural, 0=Urban |
| Educational level | 1=Illiteracy, 2=Primary school, 3=Junior high school, 4=Senior high schooland above |
| Marital status | 1=Married, 0=Unmarried |
| Number of children | 0= No children, 1= one, 2= two, 3= three or more |
| Region | 0=Eastern, 1=Central, 3=Western |
| Healthy behavior | |
| Chronic diseases history | hypertension, diabetes mellitus, cancer, pulmonary disease, cardiovascular disease, stroke, arthritis/rheumatism, dyslipidemia, hepatic disease, renal disease, gastrointestinal disorders, asthma, memory-related disorders, or psychiatric/emotional conditions. (1=Yes, 0=No) |
| Wear glasses | 1=Yes, 0=No |
| Observe the situation in the distance | 1=Very poor to 5=excellent |
| Observe the situation up close | 1=Very poor to 5=excellent |
| Worn hearing aids | 1=Yes, 0=No |
| Hearing ability | 1=Very poor to 5=excellent |
| Tooth loss | 1=Yes, 0=No |
| Hip fracture | 1=Yes, 0=No |
| Pain localization | self-reported pain in head, shoulder, arm, wrist, fingers, chest, stomach, back, waist, buttocks, legs, ankles, toes, or neck (1=Yes 0=No) |
| Sleep time | hours/day |
| Heavy exercise | 1=Yes, 0=No |
| Moderate exercise | 1=Yes, 0=No |
| Mild exercise | 1=Yes, 0=No |
| Social activities | Does Activi visit homes, play mahjong, provide free assistance, dance, participate in club activities, volunteer activities, attend school and training, take care of others, trade stocks, surf the internet, or engage in other social activities (1=Yes, 0=No) |
| History of alcohol consumption | 1=Yes, 0=No |
| Current alcohol consumption | 1=Yes, 0=No |
| Smoked in the pas | 1=Yes, 0=No |
| Smoke now | 1=Yes, 0=No |
| Subjective perception | |
| Episodic memory | Immediate recall test of 10 target words (0-10) |
| Cognitive status | Composite score (0-11) assessing temporal orientation, arithmetic reasoning, and visuospatial ability |
| Life satisfaction | 1=Very dissatisfied to 5=very satisfied |
| Self-rated health | 1=Very poor to 5=excellent |
| Health care and insurance | |
| Medical insurance types | 1=Urban employees, 2=Urban and rural residents, 3=Other insurance, 0=No insurance |
| Whether it is outpatient or inpatient | 1=Yes, 0=No |
| Number and duration of hospitalizations |  |
| Outpatient frequency |  |
| Hospitalization expenses | out of pocket expenses/total expenses (take logarithm) |
| Outpatient expenses | out of pocket expenses/total expenses (take logarithm) |
| Pension insurance | 1=Yes, 0=No |
| Whether to retire or not | 1=Yes,0=No |

**Supplementary Table 2.** Final hyperparameter settings for ML models.

| ML Model | Hyperparameter settings |
| --- | --- |
| LR | 'classifier__C': 0.01, 'classifier__class_weight': 'balanced', 'classifier__solver': 'liblinear' |
| HistGBM | 'classifier__l2_regularization': 0.5, 'classifier__learning_rate': 0.05, 'classifier__max_depth': 3, 'classifier__max_iter': 200, 'classifier__min_samples_leaf': 20 |
| MLP | 'classifier__alpha': 0.01, 'classifier__batch_size': 64, 'classifier__hidden_layer_sizes': (30, 15), 'classifier__learning_rate_init': 0.001 |
| XGBoost | 'classifier__learning_rate': 0.05, 'classifier__max_depth': 5, 'classifier__n_estimators': 50, 'classifier__subsample': 0.8 |
| Bagging | 'classifier__bootstrap': False, 'classifier__max_features': 0.6, 'classifier__max_samples': 0.6, 'classifier__n_estimators': 50 |
| DT | 'classifier__ccp_alpha': 0.01, 'classifier__max_depth': 8, 'classifier__min_samples_split': 20 |
| LightGBM | 'classifier__learning_rate': 0.01, 'classifier__n_estimators': 50, 'classifier__num_leaves': 63 |
| RF | 'classifier__max_depth': 8, 'classifier__max_features': 0.7, 'classifier__max_samples': 0.8, 'classifier__min_samples_split': 20, 'classifier__n_estimators': 100 |
| SVM | 'classifier__C': 1, 'classifier__gamma': 'scale', 'classifier__kernel': 'rbf' |
| CatBoost | 'classifier__depth': 6, 'classifier__iterations': 50, 'classifier__learning_rate': 0.05 |
